# Meta-Analysis of the Clinical Efficacy of Auricular Acupressure on Patients with Depression

**DOI:** 10.31083/AP38776

**Published:** 2025-02-28

**Authors:** Xiaohua Yang, Qingqing Liu, Xiaoping Wu

**Affiliations:** ^1^Department of Nursing, The Fourth Affiliated Hospital of School of Medicine, and International School of Medicine, International Institutes of Medicine, Zhejiang University, 322000 Yiwu, Zhejiang, China

**Keywords:** auricular acupressure, depression, quality of life, meta-analysis

## Abstract

**Objective:**

A systematic review of evaluating the clinical effects of auricular acupressure on patients with depression.

**Methods:**

A comprehensive literature search was conducted in various electronic databases including PubMed, Web of Science, Cochrane Library, Google Scholar, CNKI, Wanfang, Embase, and VIP. The search time limit was from the establishment of the database to December, 2023. The included results were integrated and analyzed, and ReviewManager 5.4 was used for meta-analysis.

**Results:**

A total of 13 studies with a total of 504 depression patients were included. Compared with the control group, auricular acupressure showed a significant reduction in depression scores, as reflected in the Hamilton Depression Scale (standardized mean difference [SMD] = –1.50, 95% confidence interval [CI]: –2.25~–0.75); risk rate [RR] = 1.96, 95% CI: 0.66~5.82), Self-Rating Depression Scale (SMD = –0.91, 95% CI: –1.15~–0.67), and Patient Health Questionnaire scores (SMD = –0.94, 95% CI: –1.46~–0.41; all *p* < 0.01).

**Conclusions:**

The meta-analysis suggested that auricular acupoint therapy is safe and effective in treating depression, and can reduce Hamilton Depression Scale (HAMD) and Pittsburgh Sleep Quality Index (PSQI) scores. There were no obvious adverse reactions. This therapy could therefore be used as a complementary therapeutic approach for patients with depression.

## Main Points

1. In the 13 studies included in this meta-analysis, the auricular acupressure group showed significant improvement in depression symptoms across all scores. This result indicates that auricular acupressure has a notable antidepressant effect in clinical practice.

2. The study results indicated that none of the included studies reported significant adverse reactions, proving that auricular acupressure is a safe and non-invasive treatment method. Compared with traditional drug therapy and other physical treatments, auricular acupressure offers a low-risk alternative.

3. This meta-analysis combined data from multiple depression assessment tools including the Hamilton Depression Scale (HAMD), Self-Rating Depression Scale (SDS), and Patient Health Questionnaire (PHQ-9). In all these different scale evaluations, the auricular acupressure group showed significant improvement in depression symptoms. This consistent verification across multiple tools enhances the reliability and generalizability of the study results, providing robust evidence for auricular acupressure as a complementary treatment for depression.

## 1. Introduction

Depression refers to a type of affective mental disorder characterized by significant and persistent low mood, reduced activity ability, and slow thinking and cognitive functions [1]. According to statistics from the World Health Organization (WHO), more than 350 million people worldwide suffer from depression, and the number of patients has increased by approximately 18% in the past 10 years. Five percent of adults worldwide suffer from depression every year, and depression has become a common disease worldwide [2, 3]. Data from the China Mental Health Survey (CMHS) in 31 provinces and cities in China from 2012 to 2015 show that depressive disorder among adults in China has a lifetime prevalence rate of 6.8%; 8.0% for women and 5.0% for men [4]. In addition, the suicide mortality rate for people with depression is more than 10 times that of the general population [5]. The incidence rate, disability rate, and mortality rate of depression are high and continue to rise, placing a huge disease burden on society and families. Research shows that there is a bidirectional relationship between insomnia and depression [6, 7].

Epidemiological studies show that 40% to 92% of insomnia symptoms are caused by mental illness. About one third of people worldwide have insomnia symptoms and daytime dysfunction, and more than 70% of depressed people also have insomnia symptoms [8]. There are many treatment methods for depression in clinical practice, including antidepressants, psychotherapy, and physical therapy. However, due to an insufficient number of psychotherapists and high treatment costs, psychotherapy is not widely used, especially in low-income countries. Psychotherapy can also cause a series of problems and may lead to secondary damage to patients [9]. The treatment process may exacerbate the patient’s self-hatred owing to events he/she experienced. In addition, clinical practice also reported adverse events related to drug treatment, such as loss of appetite, drowsiness, dizziness, nausea, and vomiting, and a risk of cardiovascular and cerebrovascular incidents [10]. The above methods therefore have certain limitations in the treatment of depression. Physical therapy may also induce conditions such as epilepsy [11]. Therefore, clinical guidance needs to explore an effective complementary and alternative therapy for depression to alleviate symptoms and cure the disease.

Acupoint massage originated from traditional Chinese medicine (TCM) and is a non-invasive technique based on TCM meridian theory that uses slow and even pressure applied to acupoints rather than needles. Auricular acupuncture therapy is therefore very effective in promoting sleep, and its efficacy in treating depression is gradually emerging [12]. Moreover, this therapy has long-lasting effects, is easy to operate, is economical and cheap, and causes few adverse reactions. Previous systematic reviews have shown that the overall effectiveness of auricular acupoint therapy combined with other therapies in the treatment of depression is better than the use of other therapies alone [11]. While TCM auricular acupuncture continues to develop in China, it has also attracted widespread attention around the world. The French physician Paul Nogier published “Auricular Point Distribution Map Shaped Like an Embryo Reflection” in the *German Journal of Acupuncture* in 1957 [13], which promoted worldwide attention to auricular point therapy. Furthermore, it stimulated the enthusiasm of scholars at home and abroad for research on auricular acupuncture therapy. Based on original research results, Chinese researchers have further carried out a large number of experimental studies, which has led to the continuous progress and development of related research on auricular acupuncture therapy.

Because of its significant efficacy, auricular acupuncture has received widespread attention from relevant scholars. Since there are currently very few systematic reviews of auricular acupuncture for the treatment of depression, this study included clinical randomized controlled trial (RCT) literature on auricular acupuncture for the treatment of depression, at home and abroad. According to the requirements and standards of the Cochrane International Collaboration Network on Evidence-Based Medicine, the effectiveness and safety of auricular acupoint therapy in treating depression were quantitatively evaluated, and its principles of action were analyzed and summarized for a meta-analysis, in order to provide evidence-based medical evidence and a more comprehensive and objective basis for auricular acupoint therapy to treat depression.

## 2. Data and Methods

### 2.1 Literature Search Strategy

Specific and systematic searches were carried out in the PubMed, Embase, Web of Science, Google Scholar, CNKI, Wanfang, and VIP databases. The search terms were: “depressive disorder” or “ear acupuncture” or “auricular acupuncture” or “ear acupressure” or “auricular acupressure” or “ear seed” or “auricular seed” or “ear pellet” or “auricular pellet” or “auriculotherapy”. The search expressions were: “treatment auricular acupuncture depressive disorder”. The search time limit was from the establishment of the database to December, 2023, and the results were limited to clinical research, not restricted by language or participant race. Manual searches were performed by reading relevant works and summarizing references. Search strategies were adjusted to comply with the relevant regulations in each database.

### 2.2 Literature Inclusion Criteria

(1) All types of Randomized Controlled Trial (RCT) (single-blind, double-blind, or non-blinded).

(2) The trial included a parallel control group and an experimental group.

(3) Research subjects were not limited by race, nationality, or disease course.

(4) Research subjects were patients with depression and insomnia, according to the Chinese Classification and Diagnostic Criteria for Mental Disorders, 3rd Edition (CCMD-3) [14] and the Classification of Mental and Behavioral Disorders (ICD-10) [15]. Depression is described in the 4th and 5th editions of the American Diagnostic and Statistical Manual of Mental Disorders [16]. Pittsburgh Sleep Quality Index (PSQI) score ≥7 points [17]. Compliance with the description of depression syndrome in the Internal Medicine of Traditional Chinese Medicine [18] and Standards for Diagnosis and Treatment of Diseases and Syndrome of Traditional Chinese Medicine and Selection of Prescriptions [19].

(5) No statistical difference in age, gender, condition, etc. of the study subjects, and the baseline consistency is good and comparable.

(6) Intervention measures: the control group was given conventional antidepressant drugs and/or sleep aids, and the experimental group was given auricular acupuncture treatment based on the control group.

(7) Outcome measures included depression and sleeplessness or quality of life, such as the Hamilton Depression Rating Scale (HAMD-24) score, Self-Rating Depression Scale (SDS) score, and PSQI score, etc.

### 2.3 Literature Exclusion Criteria

(1) Non-randomized trials. (2) Duplicate publications or data duplication. (3) Studies without a control group. (4) Animal experiments. (5) Research methods, results, and conclusions that could not be explained or did not correspond to each other. (6) Statistical methods and data analysis that had obvious errors. (7) Studies with imperfect experimental design. (8) Studies for which data could not be extracted or data were incomplete. (9) The same author has published literature in the same direction, and the best ones will be used. (10) There were no identical outcome indicators. (11) Patients with serious diseases such as of the heart, liver, kidney, brain, and blood system are included. (12) Non-Chinese and English literature.

### 2.4 Literature Screening and Data Extraction

Literature screening: two researchers independently screened the literature based on the inclusion and exclusion criteria, targeting titles and abstracts, including primary screening, secondary screening, and cross-checking to determine possible relevant studies. Noteexpress (NoteExpress v4.1.0., Beijing Aegean Lezhi Technology Co., Ltd, Beijing, China) was used to import all retrieved documents to check for duplicate material. Preliminary screening was carried out by reading the title and abstract, and then downloading and reading the full text. Firstly, a preliminary screening was carried out by reading and analyzing the titles and abstracts of the articles. Literature not satisfying the inclusion criteria or duplicate studies were removed. Secondly, re-screening included reading the full text of the article obtained from the primary screening, and then a further screening was made against the inclusion criteria. Finally, the articles were cross-checked. For documents with incomplete or questionable information, it was necessary to contact the corresponding authors for detailed information. A decision was then made whether to include the article in the study. If two researchers had different opinions on some articles, they discussed them until a consensus was reached. If no consensus could be reached, a third researcher made the final decision. Selected articles were compiled into a table for extraction and summary.

Data extraction: the extracted data mainly included title, author, publication time, grouping method, sample size, intervention measures, treatment courses, and outcome indicators, etc.

### 2.5 Quality Evaluation

Two researchers used the Cochrane risk assessment tool to conduct an item-by-item evaluation of each included study, according to the following 6 evaluation criteria:

(1) Random sequence generation

(2) Allocation concealment

(3) Blinding of participants and personnel

(4) Blinding of outcome assessment

(5) Incomplete outcome data

(6) Selective reporting

Each aspect was evaluated using three levels: low risk, unclear, and high risk. If there were any objections, the researchers discussed and negotiated among themselves or left it to a third party for a decision until a consensus was reached.

### 2.6 Statistical Analyses

All analyses were pooled using RevMan 5.4 statistical software (The Nordic Cochrane Centre, Copenhagen, Denmark), with weighted mean differences (WMD) and 95% confidence intervals (CI) for continuous data and relative risk (RR) and 95% CI for dichotomous data. The heterogeneity index (I^2^) was used to evaluate the heterogeneity of the treatment effect. When there was no significant heterogeneity among the studies (I^2^ < 50%), the fixed effect model was used. When there was significant heterogeneity among the studies (I^2^ ≥ 50%), a random effects model was used. Sensitivity analysis was performed on factors that may cause heterogeneity and literature with high sensitivity was excluded. A descriptive analysis was performed for those that could not be included in the meta-analysis.

## 3. Results

### 3.1 Literature Search

**Fig. 1.**
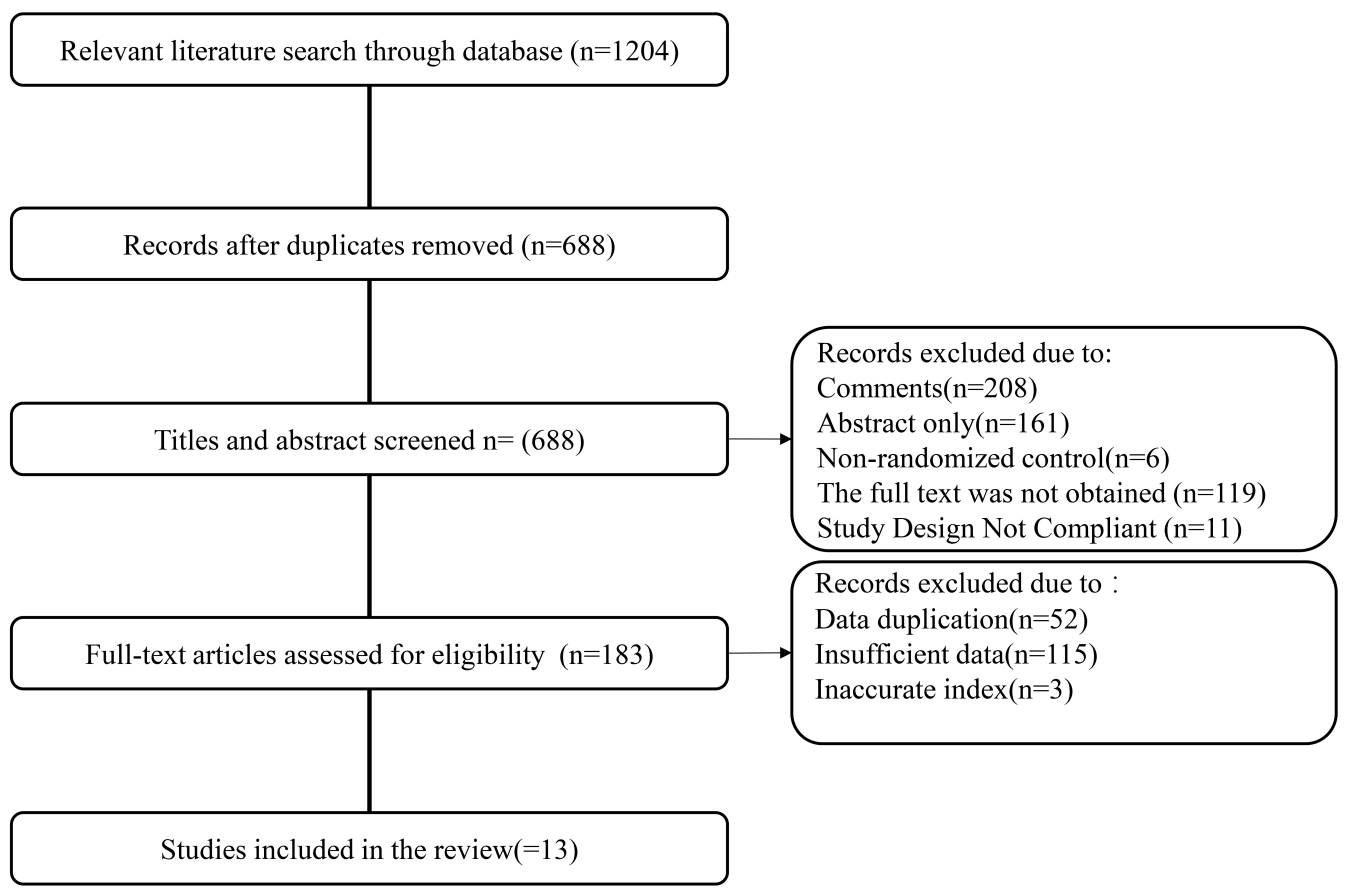
**Flow chart outlining literature search process**.

Original literature on depression and auricular acupressure, etc., published in databases such as CNKI, Wanfang, VIP, EMBASE, Web of Science, and PubMed were systematically retrieved using subject headings combined with free words resulting in 1204 articles. Of these, 375 articles that were comments or abstracts only or were non-randomized controls were eliminated, and 313 articles were retained. After reading the title and abstract, 130 articles for which the full text could not be obtained or that had incomplete experimental designs were eliminated. Finally, 13 articles were included [12, 20, 21, 22, 23, 24, 25, 26, 27, 28, 29, 30, 31]. The literature screening process is outlined in Fig. 1.

### 3.2 Basic Characteristics and Quality Evaluation of Included Literature

The demographic characteristics and baseline characteristics of the patients are shown in Table 1 (Ref. [12, 20, 21, 22, 23, 24, 25, 26, 27, 28, 29, 30, 31]).

**Table 1.** **Basic characteristics and Jadad score of included studies**.

First researcher	Number of Cases	Interventions		Curative Time (weeks)	Efficacy Index
	Investigation Group/Control Group	Treatment Group	Control Group		
DM de Oliveira Rodrigues 2023 [12]	37/37	Specific auricular acupuncture and usual care	Nonspecific auricular acupuncture and usual care	6	①
Bomi Kim 2023 [20]	23/23	auricular acupressure five acupoints (superior triangular fossa, Shenmen, kidney, heart, and occiput)	auricular acupressure (anus, knee, chest, jaw, and tooth)	8	①
Yan Shuo 2022 [21]	55/55	Auricular pressure with seeds+conventional antidepressant medications	Conventional antidepressant medications	4	①②
Xiao-Jun Yin 2022 [22]	30/30	Auricular therapist with ear seeds	Acupressure treatment on Knee, Lumbosacral Vertebrae, Shoulder, Eye, and Vision 1auricular points	4	②③④
Yen-Ting Tseng 2021 [23]	24/23	Patches with magnetic beads were pasted onto the auricular Shenmen acupoints	Blank patches with magnetic beads were pasted onto the auricular Shenmen acupoints	2	⑤
Se-Na Lee 2021 [24]	28/26	Auricular acupressure using vaccaria seeds on Shenmen, heart, occiput, anterior lobe point	Placebo auricular acupressure using vaccaria seeds on wrist, hips, elbow, shoulder point	5	⑥
Huang, Wei Ling 2019 [25]	10/5	Auricular Acupuncture: Apex	Conventional antidepressant treatment	-	⑦
		Ear Bloodletting			
de Lorent Lukas 2016 [26]	90/72	auricular acupuncture on point 51 (Sympathetic point), point 55 (Shen Men), point 95 (Kidney point), point 97 (Liver point), and point 101 (Lung point)	Progressive muscle relaxation	4	⑧
Fu Yijun 2015 [27]	55/52	Auricular therapist with ear seeds+conventional antidepressant medications	Conventional antidepressant medications	4	②
Chen Linfang 2014 [28]	27/27	Auricular therapist with ear seeds+conventional antidepressant medications	Conventional antidepressant medications	8	①②
Turan Set 2014 [29]	24/30	Six ear acupuncture sessions	Conventional antidepressant medications	12	⑥
Fan Chun 2011 [30]	33/33	Auricular therapist with ear seeds	No preventive measures are implemented	8	②
FU Wen-bin 2009 [31]	176/88	acupuncture at Siguan Points, i.e., bilateral Hegu (LI 4) and Taichong (LR 3), Baihui (GV 20) and Yintang (EX-HN3) plus ear-acupuncture	Acupuncture at non-acupoints as acupuncture placebo	12	③

Note: ① Patient Health Questionnaire-9 (PHQ-9)/PSQI-K, ② Hamilton Depression Scale (HAMD), ③ Self-rating Depression Scale (SDS), ④ Quality of Life Scale (QOL), ⑤ Geriatric Depression Scale (GDS), ⑥ Beck Depression Inventory (BDI), ⑦ Hospital Depression Evaluation Scale (HDES), ⑧ visual analog scale (VAS). The investigation group and the control group are based on the groups included in the literature. The group that received auricular acupuncture treatment is the investigation group, while the control group did not receive auricular acupoint treatment.

### 3.3 Risk of Bias

**Fig. 2.**
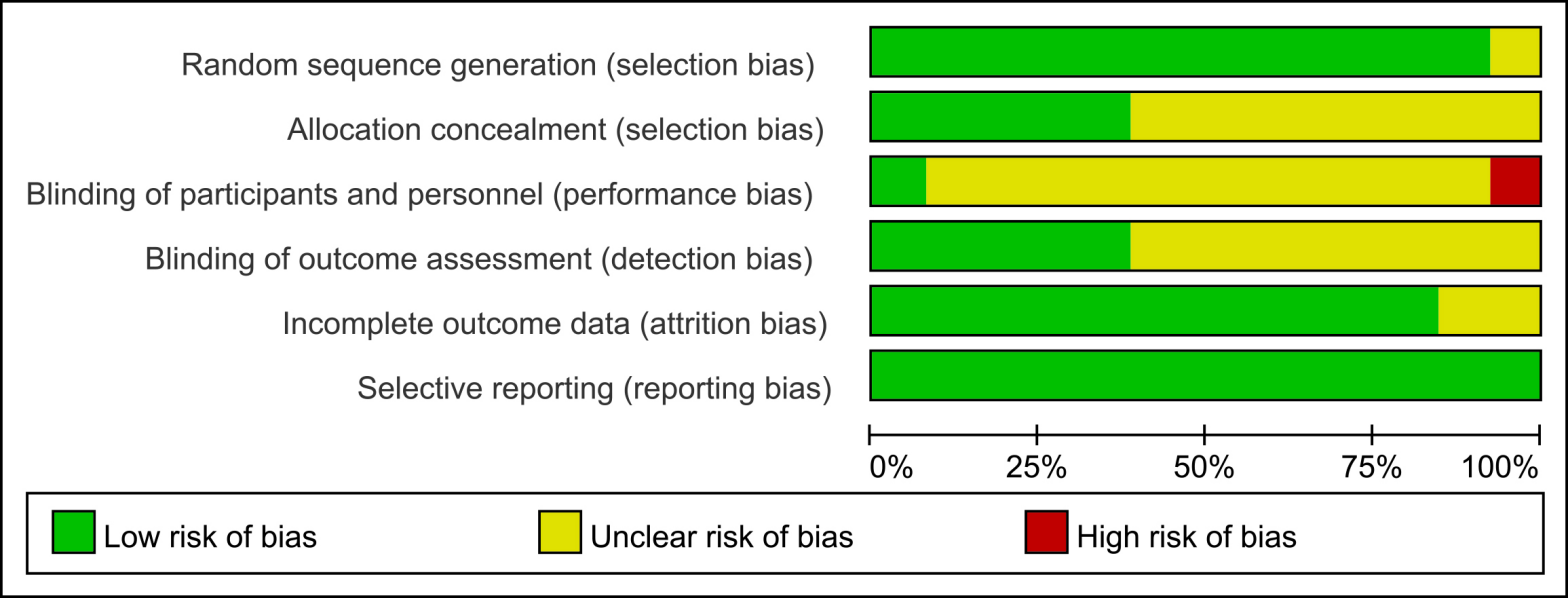
**Risk of bias bar plot**.

**Fig. 3.**
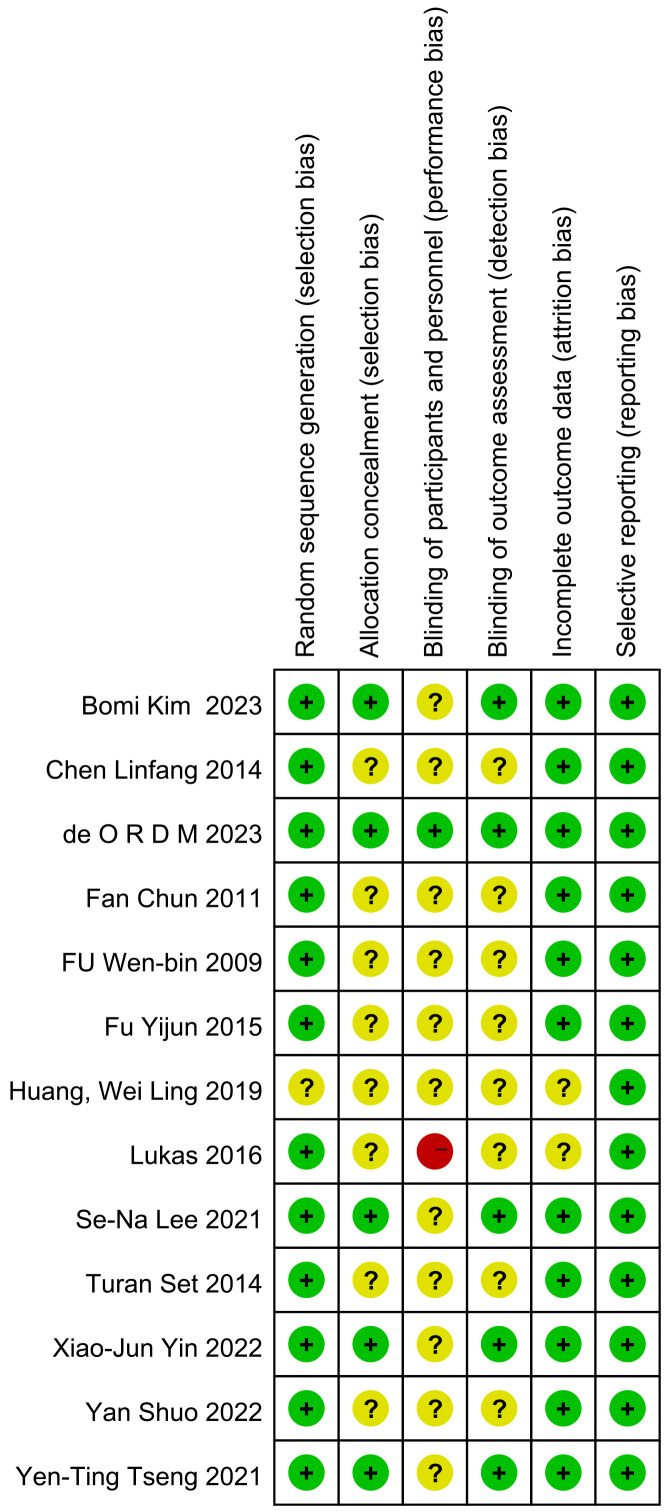
**Risk of bias summary**. +, low risk of bias; ?, unclear risk of bias; -, high risk of bias.

In order to assess the risk of bias, we used the Cochrane risk assessment tool to conduct an item-by-item evaluation of each included study. Analysis of the risk results (Figs. 2,3) showed that all the research included in this study describes the generation of random sequences. Some studies lacked clinical data after the follow-up survey. As for the hidden distribution, most studies did not describe a very comprehensive description of the embodiment-participants’ double blindness. Only the study of de Oliveira Rodrigues D M *et al*. [12] had a comprehensive and detailed description of six indicators.

### 3.4 Patient Health Questionnaire

**Fig. 4.**
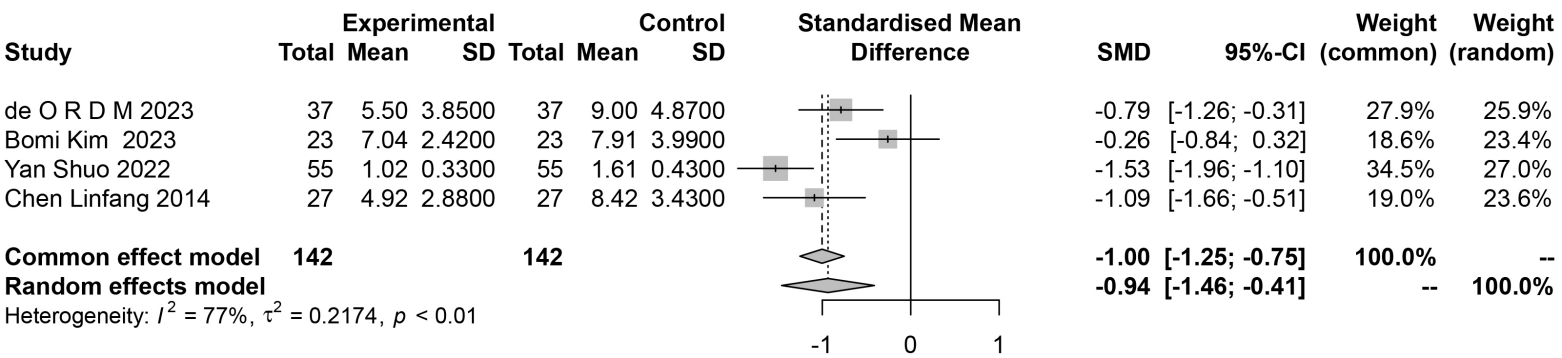
**Forest plot of the effect of depression after auricular acupuncture treatment**. SD, Standard Deviation; SMD, Standard Mean Difference; CI, Confidence Interval.

**Fig. 5.**
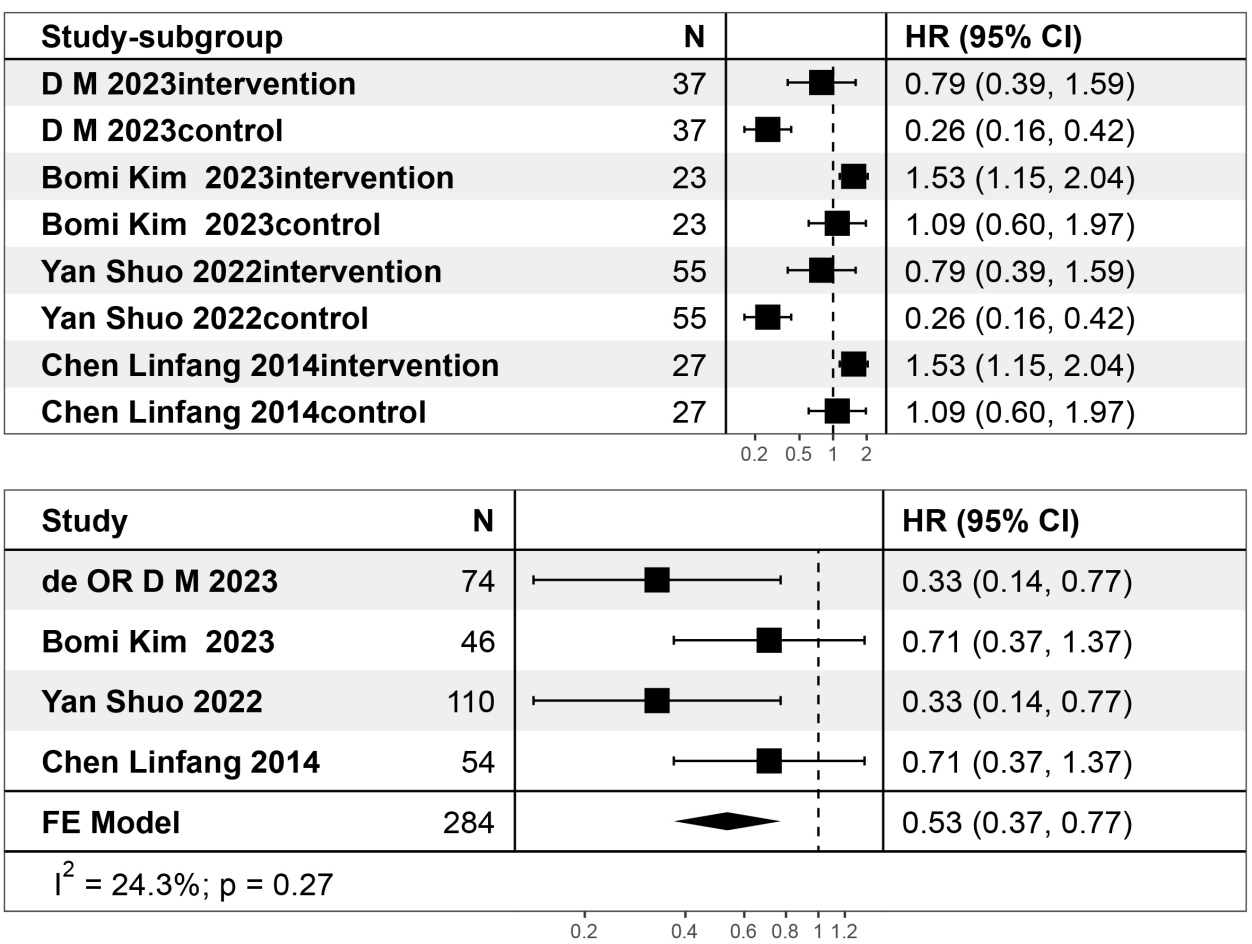
**Forest plot of subgroup analysis of Patient Health Questionnaire scores**. Hazard ratio (HR): 0.53, 95% confidence interval (CI): 0.37~0.77. Heterogeneity test: I^2^ = 24.3%.

As shown in Table 1, a total of four studies [12, 20, 21, 28] examined the improvement of patients’ depression after auricular acupuncture treatment based on changes in Patient Health Questionnaire scores. As shown in Fig. 4, the level of depression in patients after auricular acupuncture treatment was reduced (standardized mean difference (SMD) = –0.94, 95% CI: –1.46~–0.41). The results of the heterogeneity test showed that I^2^ = 77%. After performing relevant subgroup analysis (Fig. 5), I^2^ = 24.3%. Subgroup analysis can further determine the worth of testing, which helps further improve the results of the research. Heterogeneity was shown to be very low, indicating that the meta-analysis results are reliable.

### 3.5 Hamilton Depression Scale

**Fig. 6.**
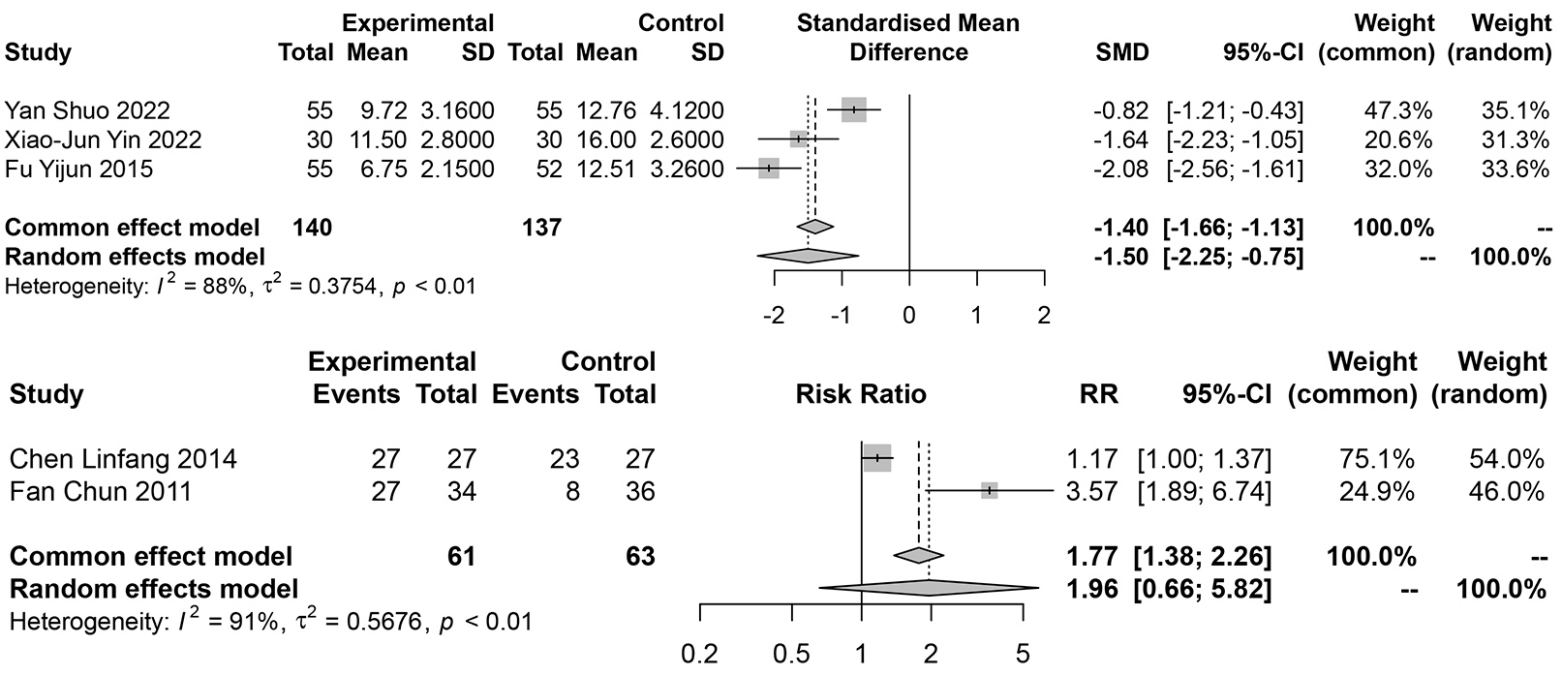
**Meta-analysis forest plot of Hamilton Depression Scale scores**. RR, Relative Risk.

**Fig. 7.**
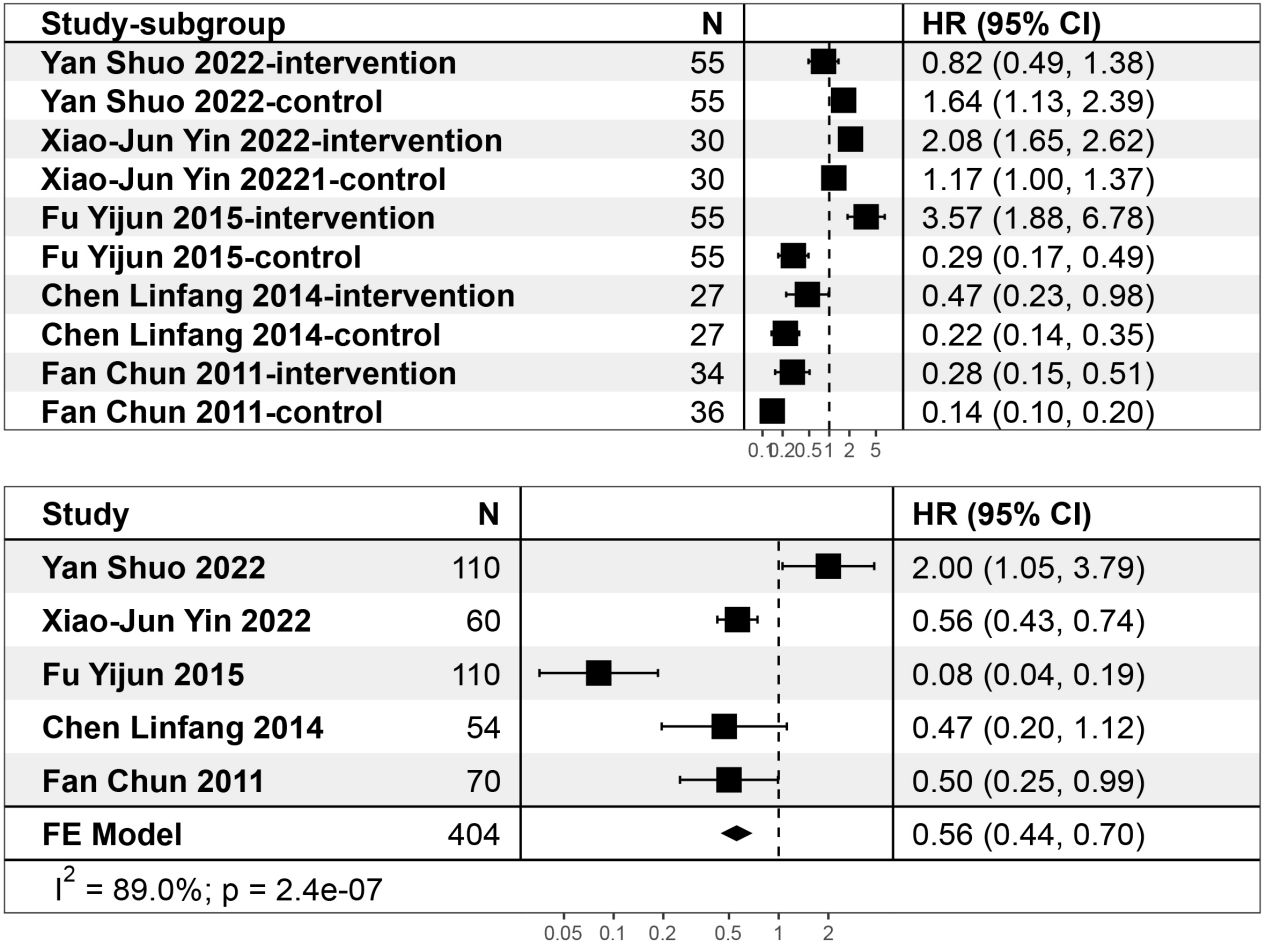
**Subgroup analysis results**.

Five studies [21, 22, 27, 28, 30] specifically described the use of the Hamilton Depression Scale to examine changes in two groups of patients with depression following different treatments. As the evaluation standards of each research institute were different, some studies were compared based on the mean standard deviation, and some studies were analyzed based on the improvement of scores. Therefore, two meta-analyses were needed. The results showed that the patients’ depression level was significantly reduced in the experimental group (SMD = –1.50, 95% CI: –2.25~–0.75) (RR = 1.96, 95% CI: 0.66~5.82) (Fig. 6), ss shown in Fig. 6. The heterogeneity test results showed that I^2^ = 88% and 91%, by relevant subgroup analysis (Fig. 7). Sensitivity analysis was performed by excluding files one by one. After excluding one study [27], the heterogeneity was 0%, indicating that this article was the source of high heterogeneity.

### 3.6 Self-Rating Depression Scale

**Fig. 8.**
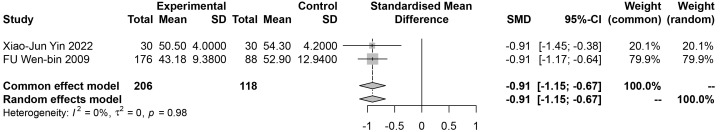
**Meta-analysis forest plot of Self-rating Depression Scale**.

Two studies specifically described the use of the Self-Rating Depression Scale to detect changes in two groups of patients with depression after different treatments. The results showed that the patients’ depression level was significantly reduced in the experimental group (SMD = –0.91, 95% CI: –1.15~–0.67) (Fig. 8). Auricular acupoint therapy improved patients’ depression, as shown in Fig. 8. The heterogeneity test results showed that I^2^ = 0%, indicating that the meta-analysis results are reliable.

### 3.7 Other Scale Score Comparisons

**Fig. 9.**
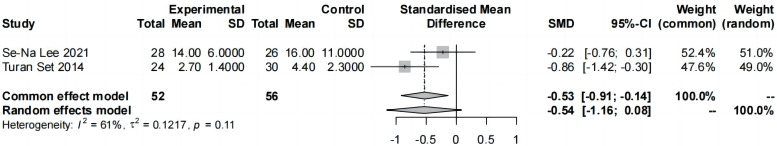
**Meta-analysis forest plot of the change in the BDI scale scores in depression patients**.

Five studies used different scales to examine the changes in two groups of depressed patients after auricular therapy. The results showed that the Beck Depression Inventory (BDI) scale score were not significantly different between groups (Fig. 9). One study [22] described that auricular acupoint therapy not only improved the patient’s emotional condition, but also improved the patient’s quality of life; confirming again that auricular acupoint therapy can improve patients’ depression.

**Fig. 10.**
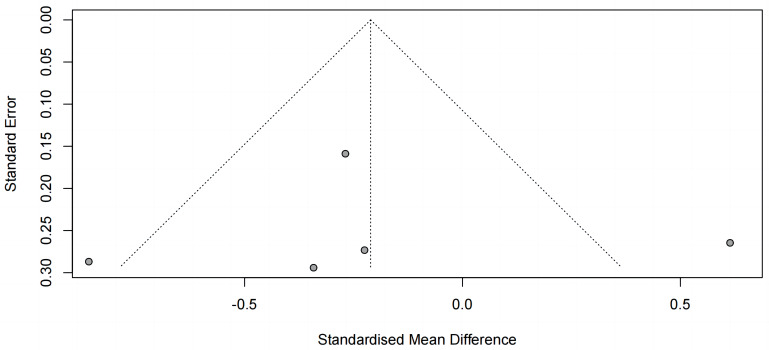
**Funnel plot of changes in other scales in patients with depression**.

Funnel plot analysis revealed significant asymmetry, suggesting potential influences, such as small sample effects or publication bias, affecting the reporting of findings (Fig. 10).

## 4. Discussion

The results of this analysis show that auricular acupoint therapy provides beneficial clinical effects in alleviating symptoms of clinical depression when compared with adjuncts to conventional treatments. In addition, it has fewer adverse effects and can also reduce HAMD and PSQI scores. The therapy is also simple, cheap, and highly accepted by patients. Although the level of evidence is moderate, acupressure shows promise as a potential treatment for depression.

The pathogenesis of depression is very complex, and the classic neurotransmitter theory still occupies a dominant position. However, new theories that include the interaction between genes and the environment, the neuroendocrine system, immune inflammation, neuroplasticity, brain structural changes, and brain functional circuitry are continuously being proposed [32]. Currently, the main clinical treatments for depression are antidepressants, tricyclic antidepressants, dopamine selective inhibitors, and 5-hydroxytryptamine receptor antagonists, etc. [33], supplemented by systemic treatments such as psychotherapy and physical therapy. Modern medicine is developing vigorously. Under ideal conditions, the cure rate for patients with depression can reach 70% to 80% after systemic treatment. However, the fact is that only 20% to 30% of depression patients in China have received a diagnosis and treatment [34], although most patients only choose drug treatment. Drug treatment has a slow onset of effect, many adverse reactions, and it is easy to relapse when the drug is discontinued. The above reasons lead to a low cure rate for depression in China. In addition, even patients who are cured of depression are still more likely to have residual symptoms. Insomnia is one of the main residual symptoms of depression, which leads to a vicious cycle of depression and insomnia [35].

Depression belongs to the categories of “depressive disease”, “insomnia”, “forgetfulness”, “epileptic syndrome”, “lily disease”, and “plum core qi” in TCM [36]. Emotional factors are the causative factors of depression, and “weak internal qi” is an important internal factor in the onset of depression. In the beginning, it is more common to have syndromes of excess or deficiency and, as time goes by, syndromes of both deficiency and excess are common. TCM treatment is based on syndrome differentiation. For example, for kidney deficiency and liver stagnation, Zi Shui Qing Gan Yin is used. For liver stagnation and spleen deficiency, Xiaoyao Powder combined with Banxia Houpu Decoction is used. The suitable techniques of TCM are mainly acupuncture, moxibustion, massage, aromatherapy, and five-tone therapy [37]. Auricular acupoint therapy belongs to the category of acupuncture. There is no acupuncture pain during auricular acupoint therapy, which makes it more adaptable and easier to accept for the patient. Auricular acupoint therapy can simultaneously improve the patient’s depression and insomnia. With the continuous deepening of the integration of traditional Chinese and Western medicine, auricular acupuncture therapy has also been innovatively developed. The innovative research team of the China Academy of Chinese Medical Sciences confirmed the existence of projection fibers from the auricular branch of the vagus nerve directly to the nucleus of the solitary tract through nerve tracing technology. They also innovatively proposed the theory of “auricular point-vagus nerve connection” and developed the transcutaneous auricular vagus nerve stimulation (taVNS) method, which provided a novel solution for auricular point therapy to treat depression and insomnia [38].

The results of the present study showed that auricular acupuncture therapy had advantages over only using medicine in reducing HAMD score, SDS score, PSQI score, and improving quality of life. This therapy can reduce depression and improve the quality of life of patients.

Auricular acupuncture therapy can improve the depression and sleep state of patients with depression and insomnia. In terms of modern neuroanatomical discoveries, auricular acupuncture therapy often uses acupuncture points in the “visceral representative areas” to treat depression and insomnia, that is, acupoints such as the “heart”, “liver”, “kidney”, and “Shenmen” [39]. Its distribution coincides with the vagus nerve distribution area of the ear—the concha area [40]. The concha area is the only area in the human body with vagus nerve distribution. The afferent fibers of the vagus nerve in the concha can directly project to the nucleus of the solitary tract. It then projects directly or indirectly through other brainstem structures such as the locus coeruleus, parabrachial nucleus, and raphe greater nucleus to the reticular formation, limbic system, and other brain areas closely related to emotion regulation and sleep [41]. Auricular acupoint therapy can stimulate the acupoints in the concha area through the above-mentioned pathways to improve the depression and sleep state associated with depression and insomnia. At the same time, the explanation can also be constructed through the “ear, brain, and organ-related” theory created by Rong Peijing’s team [42]. The bladder meridian circulates and connects the ears and the brain. As the saying goes, “the two ears connect the brain”, and the ears treat the brain. The heart governs the gods, and the heart area of the ear points controls the five internal organs (heart, liver, soul, spleen, lungs, and kidneys). The heart area is used to regulate the five internal organs. The brain is the house of the Yuan Shen, and the five internal organs are connected to the brain. The five internal organs are adjusted to help the brain. Shen is used in the heart, and its body is in the brain. It should be in the five internal organs, connecting the heart, brain, and internal organs into a whole. Auricular acupoint therapy stimulates the auricular acupuncture center in the auricle area, which can not only directly regulate the mind, but also regulate the brain and spirit by “connecting the two ears to the brain”. It can also coordinate or indirectly stimulate the five internal organs in the auricle area to regulate the five internal organs and the spirit. Thereby achieving the effect of harmonizing the mind, body, and function. Auricular acupoint therapy regulates the mind, brain, and five internal organs to improve the depression and insomnia of patients with depression and insomnia.

Limitations of this study: (1) Some of the included studies did not describe specific random allocation methods, allocation concealment, and measurement bias. (2) It is difficult to implement double-blinding in auricular acupuncture therapy, and most of the included studies were not double-blinded. (3) The treatment methods of the control groups included in the studies were not uniform. As auricular acupoint therapy originated from TCM, its acceptance in countries other than China is low. Therefore, there were few studies by foreign scholars. Most foreign scholars had conducted a summary analysis on feasibility and national acceptance. Due to the large difference in years, the quality of the included studies was unequal.

## 5. Conclusions

Auricular acupuncture is very effective in treating depression. The results of this systematic review showed that auricular acupoint therapy is safe and effective in treating depression, and can reduce HAMD and PSQI scores, etc. There are no obvious adverse reactions. However, due to the limitations of the number and quality of included studies, our conclusions need to be supported by more high-quality research evidence. Moreover, this therapy has long-lasting clinical efficacy, is easy to operate, is economical and cheap, and has few adverse reactions. Therefore, it can be promoted and used clinically, based on its characteristics, in the future.

## Availability of Data and Materials

The datasets for this study are available from the corresponding author on reasonable request.

## Supplementary Material

Supplementary material associated with this article can be found, in the online version, at https://doi.org/10.31083/AP38776.
